# Transforming Growth Factor Beta Receptor 2 (TGFBR2) Changes Sialylation in the Microsatellite Unstable (MSI) Colorectal Cancer Cell Line HCT116

**DOI:** 10.1371/journal.pone.0057074

**Published:** 2013-02-27

**Authors:** Jennifer Lee, Seda Ballikaya, Kai Schönig, Claudia R. Ball, Hanno Glimm, Juergen Kopitz, Johannes Gebert

**Affiliations:** 1 Department of Applied Tumor Biology, Institute of Pathology, University Hospital Heidelberg, Heidelberg, Germany; 2 Clinical Cooperation Unit Applied Tumor Biology, German Cancer Research Center (DKFZ), Heidelberg, Germany; 3 Department of Molecular Biology, Central Institute of Mental Health, Medical Faculty Mannheim/Heidelberg University, Mannheim, Germany; 4 Department of Translational Oncology, National Center for Tumor Diseases (NCT) and German Cancer Research Center (DKFZ), Heidelberg, Germany; China Medical University, Taiwan

## Abstract

Aberrant glycosylation is a common feature of many malignancies including colorectal cancers (CRCs). About 15% of CRC show the microsatellite instability (MSI) phenotype that is associated with a high frequency of biallelic frameshift mutations in the A10 coding mononucleotide microsatellite of the *transforming growth factor beta receptor 2* (*TGFBR2*) gene. If and how impaired TGFBR2 signaling in MSI CRC cells affects cell surface glycan pattern is largely unexplored. Here, we used the TGFBR2-deficient MSI colon carcinoma cell line HCT116 as a model system. Stable clones conferring doxycycline (dox)-inducible expression of a single copy wildtype *TGFBR2* transgene were generated by recombinase-mediated cassette exchange (RMCE). In two independent clones, dox-inducible expression of wildtype TGFBR2 protein and reconstitution of its signaling function was shown. Metabolic labeling experiments using the tritiated sialic acid precursor *N*-acetyl-D-mannosamine (ManNAc) revealed a significant decline (∼30%) of its incorporation into newly synthesized sialoglycoproteins in a TGFBR2-dependent manner. In particular, we detected a significant decrease of sialylated ß1-integrin upon reconstituted TGFBR2 signaling which did not influence ß1-integrin protein turnover. Notably, TGFBR2 reconstitution did not affect the transcript levels of any of the known human sialyltransferases when examined by real-time RT- PCR analysis. These results suggest that reconstituted TGFBR2 signaling in an isogenic MSI cell line model system can modulate sialylation of cell surface proteins like ß1-integrin. Moreover, our model system will be suitable to uncover the underlying molecular mechanisms of altered MSI tumor glycobiology.

## Introduction

About 15% of hereditary and sporadic colorectal tumors display the high frequency microsatellite instability (herein termed MSI) phenotype. MSI manifests as length variations of numerous short repetitive sequences (microsatellites) caused by loss of normal DNA mismatch repair (MMR) function in these tumor cells. If MSI affects coding microsatellites of expressed genes the resulting frameshift mutations lead to premature translational termination and impaired protein function [1]–[3]. A large number of genes harboring microsatellites in their coding regions have been identified and found to be frequently affected by frameshift mutations in MSI colorectal tumors [4]–[7]. Mutations in a limited number of these genes are believed to drive MSI tumorigenesis. *TGFBR2* is one of the most interesting MSI target genes because it is part of a key signaling pathway in colon epithelial cells and found to be inactivated at high frequency by biallelic frameshift mutations in MSI colorectal tumors [8]. TGFBR2 is a transmembrane serine-threonine kinase that is capable to repress proliferation and also induces apoptosis and differentiation triggered by transforming growth factor beta (TGF-ß) activation [9]–[11]. Upon binding of the ligand TGF-ß1 the formation of a hetero-tetrameric receptor complex comprised of type 1 (TGFBR1) and type 2 (TGFBR2) receptors is initiated and downstream proteins such as SMAD2 and SMAD3 become phosphorylated [10], [12]. These phosphorylated SMAD proteins then associate with SMAD4 and upon translocation to the nucleus direct transcription of different target genes like *SMAD7* [13] or *SERPINE* [14].

Many proteins, especially cell surface proteins, are glycosylated and require glycan modifications in order to confer normal function in cell communication, recognition and adhesion. Moreover, altered cell surface glycosylation has been implicated in tumorigenesis and metastasis [15]–[17]. In colon cancer, mRNA expression of various glycosyltransferases was found to be increased when compared to corresponding normal colon mucosa [18], [19]. In addition, fucosylation of cell surface proteins has also been implicated in cancer [20]. Fucosyltransferases are associated with the formation of tumor antigens like sialyl-Le^x^ and -Le^a^ [21]. Sialic acid is a key component of glycoproteins and has been correlated with metastasis [22]. Different positioning of sialic acid on the cell surface lead to masking or unmasking of specific saccharides that are important for metastasis [23]. It has been demonstrated, that sialic acid correlates with *in vivo* tumorgenicity in the CRC cell line HCT116 [24].

ß1-Integrin is one member of a large protein family of adhesion proteins known to be highly sialylated. Integrins are glycoproteins that form hetero-dimeric complexes of α- and ß-subunits conferring different ligand affinities. Since signaling by integrins is involved in several key cellular processes like focal adhesion and motility, impaired integrin signaling has been implicated in cancer metastasis [25], [26]. It has been reported that the binding of the lectin SNA to sialylated ß1-integrin is increased in colon tumor tissue compared to normal colonic epithelium [27]. Moreover, de-sialylation of ß1-integrin was shown to stimulate its binding to glycoproteins of the extracellular matrix [28], [29].

Although TGFBR2 signaling is involved in cell-cell communication, cell adhesion and cell migration the role of this pathway in the glycosylation pattern of cell surface proteins is largely unexplored. Experimental evidence suggested a possible link between mutated MSI target genes and the glycosylation pattern at the cell surface [30]. In the present study, we used the TGFBR2-reconstituted MSI colorectal cancer cell line HCT116 as a model system to analyze TGFBR2-dependent alterations in protein glycosylation. We detected a decrease in global sialylation of newly synthesized proteins thereby inversely reflecting the increased sialylation observed in primary colorectal tumors. In particular, we identified TGFBR2 signaling as a modulator of the sialylation levels of ß1-integrin.

## Materials and Methods

### Plasmids

S2F-cLM2CG-FRT3 [31] contains a tet-controlled bidirectional transcription unit for concurrent regulation of the two reporter genes firefly *luciferase* and red fluorescent protein *mCherry.* This expression cassette is flanked by two hetero-specific FLP-recognition sites, a mutated F3 and a wildtype F site [32]. For retroviral assembly, we used the vectors pVPack-GP and pVPack-VSV-G (Stratagene). Recombination was mediated by the enzyme Flpo-recombinase that is encoded by the plasmid pCAGGS-Flpo-IRES-Puro obtained from Michael Hahn (DKFZ, Heidelberg). The plasmid pE11.F3.HygTK.F [31] encoding a hygromycin B phosphotransferase-thymidine kinase (HygTK) translational fusion protein was used for antibiotic selection and generation of the HCT116-HygTK master cell line. The retroviral vector S2F-cLM2CG-FRT3-TGFBR2 was generated by PCR amplification of the wildtype *TGFBR2* cDNA from the expression plasmid pcDNA3.1/His-TGFBR2 [30] using primers that carry *EcoRI* or *NotI* (NEB) restriction sites (Table S1) and replacement of the *EcoRI*/*NotI mCherry* fragment of S2F-cLM2CG-FRT3. Verification of the correct insertion site and sequence of the wildtype *TGFBR2* gene was confirmed by DNA sequence analysis.

### Cell Lines

All cell lines were cultured in DMEM (PAA) supplemented with 10% heat-inactivated FBS Gold (PAA) and 100 U/ml penicillin and 100 µg/ml streptomycin (PAA) using standard conditions. The parental CRC cell line HCT116 has been purchased from European Collection of Cell Cultures (ECACC). This MMR-deficient cell line exhibits the MSI phenotype and is refractory to TGF-ß-mediated signaling due to biallelic frameshift mutations in the A10 coding microsatellite of the endogenous *TGFBR2* gene. The HCT116 AWE17 (HCT116-Tet-On) cell line is a stably transfected derivative of the parental HCT116 cell line conferring constitutive expression of the reverse transcriptional transactivator (rtTA) and the EGFP protein [33]. The HepG2 cell line was used as a positive control for SMAD2 signaling. 293T cells were obtained from ATCC. For signaling experiments, cells were starved for 17 h in the presence and absence of 1 µg/ml dox (Sigma) and subsequently incubated with 10 ng/ml recombinant TGF-ß1 (Cell Signaling) for 1 h. Transfection experiments were carried out using Fugene HD Transfection Reagent according to the manufacturer‘s instruction (Roche). Antibiotic selection was performed using hygromycin B (Hyg, 100 µg/ml, PAA), puromycin (1.5 µg/ml, Sigma) and ganciclovir (Gan, 40 µM, Roche) for the indicated steps.

### Generation of HCT116-TGFBR2 Cells

**Figure 1:**
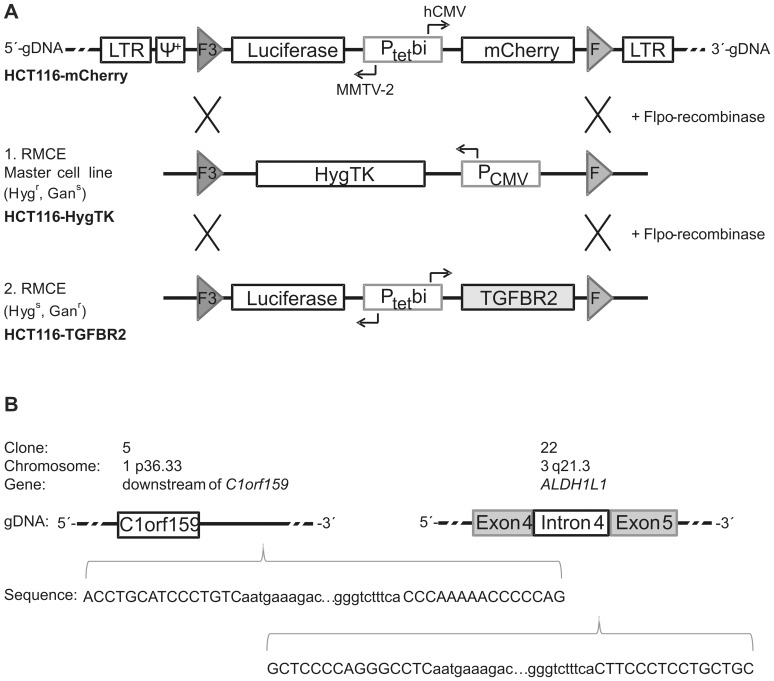
Generation of dox-inducible HCT116-TGFBR2 cell lines. (A) Schematic outline of recombination-mediated cassette exchange (RMCE). Retroviral transduction was performed using the proviral vector S2F-cLM2CG-FRT3 that contains a bidirectional dox-inducible promoter (P_tet_bi) allowing concurrent expression of two marker genes (*luciferase* and *mCherry*) in HCT116-mCherry clones. Expression cassettes are flanked by mutant (F3) and wildtype Flp-recombinase target sites (F) that allow directed cassette exchange via Flpo-recombinase. Retroviral packaging signal (Ψ^+^) and long terminal repeats (LTR) are indicated. (B) Characterization of viral integration sites by nrLAM-PCR and sequencing. In the upper part, clone-specific integration sites and affected genomic loci (open reading frame (orf); *Aldehyde dehydrogenase family 1 member L1* (*ALDH1L1*)) are depicted. In the lower part, 5′- and 3′- viral LTR DNA sequences (small letters) as well as the flanking genomic DNA sequences (capital letters) are shown.

We have applied a retroviral based approach described previously [31]. Briefly, 10^6^ 293T cells were co-transfected with 3 proviral plasmids [34], [35]. 10^5^ HCT116-Tet-On cells (∼30% confluency) were infected at a MOI of 0.01 and 0.05 to ensure single copy virus integration. Successfully transduced HCT116-Tet-On cells were induced with 0.2 µg/ml dox and mCherry-positive cells were screened and isolated by FACS (On-Off-On). Single mCherry-positive cells were selected for clonal expansion (HCT116-mCherry). In the first recombination step (RMCE), 5×10^5^ HCT116-mCherry cells were co-transfected on 6-well plates with 2 µg pE11.F3.HygTK.F plasmid carrying a Hyg-TK expression cassette flanked by two recombination sites (F3/F) and 2 µg pCAGGS-Flpo-IRES-Puro (Figure 1A). The resulting HCT116-HygTK master cell clones, which are Hyg resistant and sensitive to Gan (Hyg^r^, Gan^s^), underwent a second RMCE resulting in HCT116-TGFBR2 clones that conferred dox-inducible expression of TGFBR2 and luciferase concurrently (Figure 1A). Quantification of dox-inducible expression levels were determined by luciferase assays.

### PCR and Sequencing

Standard PCR was performed using the HOT FIREPol DNA Polymerase (Solis Biodyne) with the following cycling conditions: initial denaturation 95°C 15 min; 35 cycles of 95°C denaturation for 30 s, 60°C annealing for 30 s, 72°C elongation for 1 min and a final elongation step at 72°C for 2 min. DNA sequencing was performed using the BigDye Terminator v1.1 sequencing kit (Invitrogen). Analysis was carried out on an ABI3100 genetic analyzer (Applied Biosystems).

### Linear amplification-mediated (LAM)-PCR

Genomic DNA was extracted from the cell lines according to the manufacturer‘s protocol using DNeasy Blood & Tissue Kit (Qiagen). Non-restrictive (nr) LAM-PCR was done as previously described [36]. In brief, 1 µg of gDNA derived from transduced cell clones was used for the linear amplification of the vector genome junctions with biotinylated primer (Table S1). After enrichment of the amplified fragments via magnetic beads, the second DNA strand was generated. After ligation of a known oligonucleotide to the unknown part of the amplicons, two nested exponential PCRs were performed. For further preparation of the samples adapters were added to both ends of the amplicons by an additional exponential PCR to allow Roche 454-specific amplification and sequencing. For parallel sequencing of different samples a 6–10 bp barcode was used. 40 ng of DNA was amplified using the following PCR program: initial denaturation for 120 s at 95°C; 12 cycles at 95°C for 45 s, 58°C for 45 s and 72°C for 60 s; final elongation 300 s at 72°C. Raw LAM-PCR amplicon sequences were separated according to the introduced barcode, further trimmed and aligned to the human genome sequence using BLAT (Assembly February 2009) [37].

### Luciferase Assay

Luciferase activity was measured by the Luciferase Assay System (Promega) in duplicate according to the manufacturer‘s instruction and normalized to protein concentration determined by Bradford assay (BioRad).

### Western Blot Analysis

Cell pellets were resuspended in RIPA buffer (50 mM Tris-HCl pH 7.4, 150 mM NaCl, 1% Triton X-100, 1% sodium-deoxycholate, 0.1% SDS, 0.1 mM CaCl_2_ and 0.01 mM MgCl_2_). After sonication, incubation (1 h, 4°C) and centrifugation (12,000 g, 20 min, 4°C) protein concentration of the lysate was measured by Bradford assay. For immunoblotting, 50 µg protein was separated on 4–12% Bis-Tris Gels (NuPAGE, Invitrogen) and electroblotted onto a nitrocellulose membrane. After blocking the membrane for 30 min at room temperature (RT) in 5% skim milk/TBST (20 mM Tris-HCl pH 7.5, 0.5 M NaCl and 0.1% Tween-20), the following primary antibodies were used in blocking solution: mouse anti-TGFBR2 (sc-17799; Santa Cruz; 1∶500, 4°C, overnight); mouse anti-ß-Actin (MP Biomedicals; 1∶30,000, RT, 1 h); rabbit anti-phospho-SMAD2 (Ser465/467; Cell Signaling; 1∶1000, 4°C, overnight); rabbit anti-SMAD2 (86F7; Cell Signaling; 1∶1000, 4°C, overnight). After several washing steps (10 min each at RT) in TBST, blots were incubated with the secondary antibodies sheep anti-mouse-IgG HRP (1∶5000; GE-Healthcare) and goat anti-rabbit-IgG HRP (1∶2500; Promega) for 1 h at RT. After three washing steps (10 min each at RT) in TBST, signals were detected using Western Lightning Plus ECL (PerkinElmer).

### Real-Time RT-PCR

1 µg of total RNA was isolated with the RNeasy Kit (Qiagen) and reverse transcribed using oligo-dT primers and SuperScript II reverse transcriptase according to the manufacturer‘s protocol (Invitrogen). For real-time reverse transcription (RT)-PCR experiments, specific primers (Table S1 and S2) and PowerSYBR Green Master Mix (Applied Biosystems) were used. Triplicates of different cDNA samples (-dox versus +dox) were analyzed in the StepOnePlus thermo-cycler (Applied Biosystems) with the following program: 95°C for 10 min, followed by 40 cycles of 95°C for 15 s and 60°C for 1 min. Data were analyzed by StepOne Software v2.1 (Applied Biosystems). Gene expression was normalized to expression of the reference genes *GAPDH* and *hydroxymethylbilane synthase* (*HMBS*).

### Proliferation Assay

MTS proliferation assays were performed in triplicate with the CellTiter 96 Aqueous kit (Promega) according to the manufacturer‘s instruction.

### Incorporation of Radioactive Labeled Monosaccharides

5–10×10^4^ cells/well were seeded in triplicate on a 6-well plate. After 24 h, cells were re-fed with 2 ml new media containing 0.185 MBq of the respective ^3^H-labeled saccharide (^3^H-ManNAc (*N*-[mannosamine-6-^3^H]), [185–370 GBq/mmol] or ^3^H-L-fucose (L-6-^3^H), [1.48–2.22 TBq/mmol], American Radiolabeled Chemicals, Inc.) and 10 ng/ml TGF-ß1. Cells were grown in the presence or absence of 0.5 µg/ml dox to obtain a confluency of 60–80%. After 72 h, cells were washed 3 times with PBS and scraped off. Cells were centrifuged 5 min at 1000 g at RT and washed with PBS. The cell pellet was solubilized in 400 µl of 0.2 N NaOH for 1 h at 56°C. Protein concentration was determined by Lowry assay. 1 µg BSA and 400 µl of 10% TCA were added to precipitate proteins by centrifugation (10 min, 12,000 g, RT) and to remove unincorporated labeled saccharides. The pellet was then resuspended in 400 µl of 1 N NaOH and neutralized by 200 µl of 2.5 N acetic acid and mixed with 10 ml scintillation cocktail (Ultima Gold; PerkinElmer). The samples were counted using a liquid scintillation analyzer (TRI-CARB 2900TR; Packard) and dpm measurements were conducted with automatic quench correction applying the transformed Spectral Index of the External Standard/Automatic Efficiency Control (tSIE/AEC) method. Results were expressed as dpm and normalized to protein amount (mg).

### Radioactive Labeling and ß1-Integrin Immunoprecipitation (IP)

For dual labeling using ^35^S-L-methionine [37 TBq/mmol] (American Radiolabeled Chemicals, Inc.) and ^3^H-ManNAc, 1–2×10^6^ cells were seeded in triplicate on 10 cm plates. After 24 h, medium was replaced by 5 ml new media containing 1.11 MBq of ^3^H-ManNAc, 0.37 MBq of ^35^S-L-methionine and 10 ng/ml TGF-ß1. Cells were grown in presence or absence of 0.5 µg/ml dox. After 72 h, cells were washed 3 times with PBS and scraped off. The cells were centrifuged 5 min at 1000 g at 4°C and washed with PBS. The pellet was resuspended in 150 µl RIPA buffer. Cells were sonicated and incubated 1 h at 4°C while rotating. After centrifugation at 4°C for 30 min at 12,000 g the resulting lysate was used to determine the protein concentration by Bradford assay. For ß1-integrin IP, 1.6 mg lysate was incubated with 1.7 µg ß1-integrin antibody (P5D2 from DSHB, Iowa) in a volume of 300 µl for 2 h at 4°C. As a control for unspecific binding to the beads, each cell clone was incubated without the antibody in the presence or absence of dox and counts were subtracted from the results. 25 µl protein A/G agarose (Oncogene) were washed 3 times with 1 ml RIPA buffer and then incubated with the lysate and the antibody overnight by rotating at 4°C. The beads were washed 5 times with 500 µl RIPA buffer and eluted with 2×200 µl of 1x protein sample buffer (106 mM Tris-HCl, 141 mM Tris base, 2% SDS, 10% glycerol, 0.51 mM EDTA) at 99°C for 5 min. The samples were mixed with 10 ml of scintillation cocktail and counted by a luquid scintillation analyzer applying the tSIE/AEC method. Results were expressed as dpm. As a control for unspecific binding the elution of the IP was analyzed by SDS-PAGE and subsequent stained with SYPRO-Ruby (Invitrogen). In parallel, the corresponding bands were detected by Western blotting using a ß1-integrin antibody (Cell Signaling) (data not shown). For the pulse-chase experiment, cells were seeded and treated as described above, pulsed for 72 h with 1.11 MBq ^35^S-L-methionine/5 ml fresh media and then harvested at 5 different time points (0h, 4h, 8h, 16h, 24h, chase). Subsequently, cells were lysed as described above. 1.5 mg lysate was incubated with 1.3 µg ß1-integrin antibody in a total volume of 300 µl of RIPA buffer and rotated for 2h at 4°C. After addition of protein A/G agarose, samples were processed as described above.

## Results

### Generation of Doxycycline-inducible HCT116-mCherry Clones

In order to investigate TGFBR2-dependent changes of protein glycosylation, we sought to generate a MSI colorectal cancer cell line model system that enables inducible reconstitution of TGFBR2 expression in an isogenic background. In general, this system inversely reflects the situation of primary MSI colorectal tumors that have lost TGFBR2 expression during tumor progression. As a first step, the MSI cell line HCT116-Tet-On that constitutively expresses the dox-regulated rtTA [33] was genetically modified by transduction with self-inactivating retroviruses expressing tet-controlled luciferase and mCherry (S2F-cLM2CG-FRT3) at low multiplicity of infection (MOI) to favor single copy integration. Quantitative analysis of stable clones identified two HCT116-mCherry clones (#5 and #22) with 40–70-fold dox-regulated induction of reporter gene expression as determined by luciferase assays. Identification and molecular characterization of the retroviral integration sites by nrLAM-PCR and sequencing confirmed that only a single copy had been inserted. Integration of the *luciferase-mCherry* expression cassette was localized on chromosomes 1 (*C1orf159)* for clone #5 and on chromosome 5 (*ALDH1L1*) for clone #22, respectively (Figure 1B). More importantly, integrated expression cassettes did not alter growth of HCT116-mCherry clones when compared to their HCT116-Tet-On progenitors. In order to direct insertion and dox-inducible expression of the *TGFBR2* transgene at exactly these genomic sites, we pursued a two-step strategy (Figure 1A). In a first recombination step, the *luciferase-mCherry* cassette was replaced by an expression cassette encoding the Hyg-TK fusion protein thereby generating two master cell lines HCT116-HygTK #5 and #22 that allow integration of any gene of interest at these two genomic loci. Accordingly, we replaced the *HygTK* expression cassette in a second RMCE by a *luciferase-TGFBR2* expression cassette resulting in HCT116-TGFBR2 clones #5 and #22 that were characterized and used for subsequent analyses.

### Characterization of HCT116-TGFBR2 Clones

**Figure 2:**
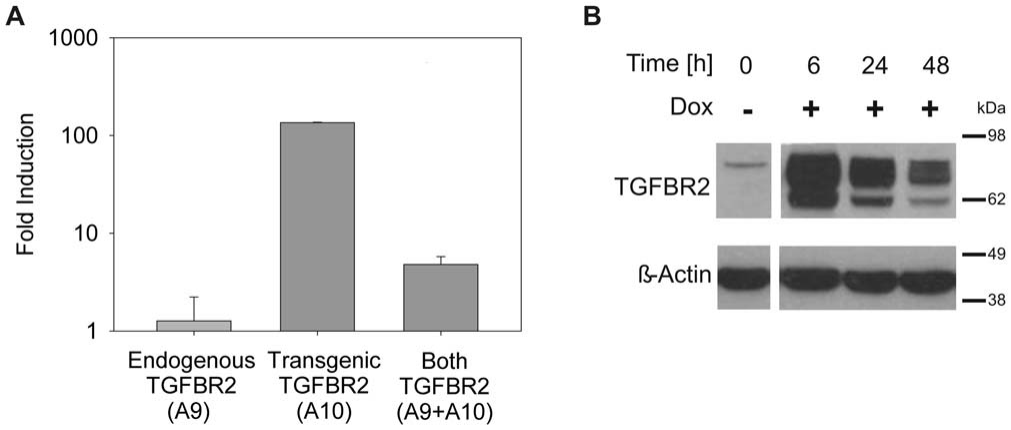
Reconstitution of TGFBR2 expression. (A) Real-time RT-PCR analysis of endogenous mutant (A9), transgenic wildtype (A10) or both *TGFBR2* transcripts in the absence and presence of dox (1 µg/ml) are shown. Results represent the mean of three independent observations ±S.D. (B) Western blot analysis, demonstrating the presence of dox-inducible (1 µg/ml) TGFBR2 expression in a time-dependent manner. ß-Actin served as a loading control. Data are shown for HCT116-TGFBR2 clone #5, but also apply to clone #22 (data not shown).

**Figure 3:**
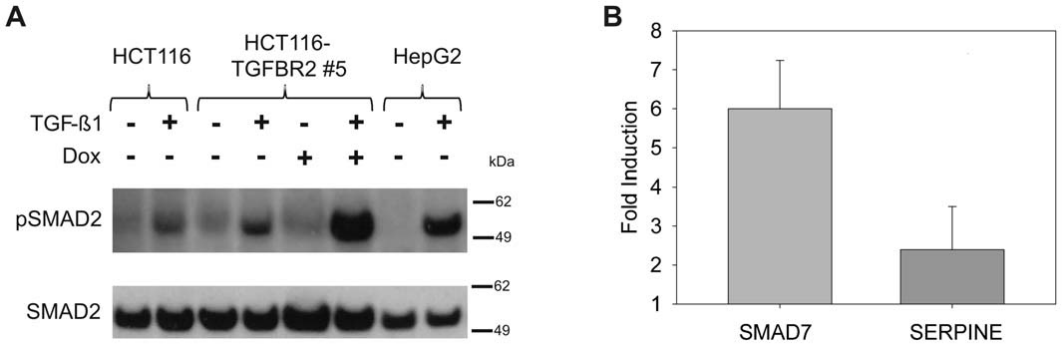
Reconstitution of TGFBR2 signaling. (A) Phosphorylation of SMAD2 (pSMAD2) was detected by Western blot analysis. Treatment with dox (1 µg/ml) and TGF-ß1 (10 ng/ml) displayed higher levels of pSMAD2 in comparison to cells grown in the absence of dox. The parental HCT116 cell line served as negative control, whereas TGF-ß1 responsive HepG2 cells were used as a positive control. Total SMAD2 has been used as a loading control. (B) Target gene transcription of TGFBR2 signaling. Real-time RT-PCR experiments revealed dox-dependent *SMAD7* and *SERPINE* upregulation. Data are shown for HCT116-TGFBR2 clone #5 but also apply to clone #22 (data not shown). Values represent the means of three independent experiments ±S.D.

Next, we analyzed these HCT116-TGFBR2 cells in more detail. Luciferase analysis revealed that reporter gene induction levels, originally obtained in HCT116-mCherry cells (40–70-fold), were also detected in HCT116-TGFBR2 cells. This excludes any effects of the RMCE-based strategy or the expression cassette used on the inducibility of our model system. Moreover, when we used transcript-specific primers in real-time RT-PCR analysis to compare the expression of the endogenous A9 mutant *TGFBR2* transcript, the transgenic A10 *TGFBR2* wildtype transcript or both transcripts upon dox exposure, no change in the endogenous *TGFBR2* transcript level was observed. Instead, dox treatment led to a strong induction of the transgenic *TGFBR2* wildtype transcript (Figure 2A). To exclude, that the reconstituted *TGFBR2* wildtype gene might have acquired similar inactivating mutations due to the lack of DNA MMR function in these cells, we sequenced transcript-specific *TGFBR2* cDNAs and identified exclusively A9 mutant or A10 wildtype repeats in the endogenous or transgenic *TGFBR2* transcripts, respectively. Thus mutational inactivation of the reconstituted *TGFBR2* transgene in these MSI and MMR-deficient HCT116-TGFBR2 clones could be excluded. In addition to these transcript analyses, we also examined the inducibility and functionality of the TGFBR2 protein by immunoblotting. In the absence of dox, no TGFBR2 protein was detected whereas in the presence of dox, specific TGFBR2 protein bands of the expected size range (75 kDa) were observed. When time course analysis was performed, TGFBR2 protein levels reached a peak within 6 h but subsequently declined within 24 to 48h (Figure 2B). For proof of functionality of the reconstituted TGFBR2 protein, we investigated its signaling ability. Accordingly, phosphorylation of SMAD2, the first downstream effector of TGFBR2 signaling, was examined by Western blot analysis (Figure 3A). In the absence of TGFBR2 expression (-dox), TGF-ß1 treatment induced a basal level of pSMAD2 in both parental HCT116-Tet-on and HCT116-TGFBR2 cells. However, when TGFBR2 expression was induced (+dox) in the presence of its ligand, TGF-ß1, a significant increase of pSMAD2 levels far beyond the basal level was observed. Moreover, we analyzed whether these *TGFBR2*-reconstituted cells are capable to regulate the transcription of several well-known TGFBR2 target genes like *SMAD7* and *SERPINE*. Real-time RT-PCR analysis revealed dox-dependent *SMAD7* and *SERPINE* upregulation and thus confirmed normal signaling activity (Figure 3B). Furthermore, proliferation is reduced when TGF-ß1 signaling is elicited in HCT116-TGFBR2 cells upon dox and TGF-ß1 treatment compared to HCT116-TGFBR2 cells exposed to TGF-ß1 in the absence of dox. However, proliferation remained unaffected among uninduced (-dox) and induced (+dox) parental HCT116-Tet-on cells or HCT116-TGFBR2 (-/+dox) cells in the absence of TGF-ß1 ligand (Figure S1A). None of these cells showed any morphological alterations (Figure S1B). Overall, these results demonstrate that both TGFBR2 clones express a functionally intact TGFBR2 protein and exhibit proper TGFBR2-mediated signaling.

### TGFBR2-dependent Glycan Alterations

**Figure 4:**
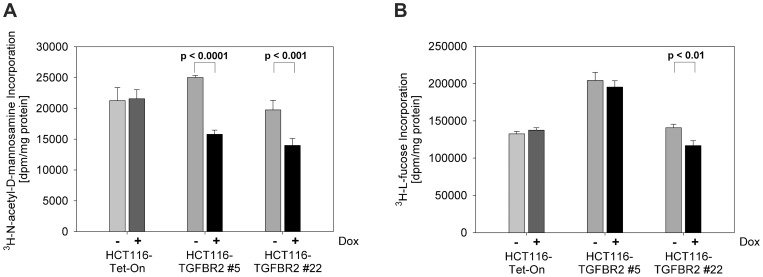
Incorporation of ^3^H-labeled monosaccharides. Radioactive labeling experiments were performed in the presence and absence of dox (0.5 µg/ml) and by exposure to TGF-ß1 (10 ng/ml) for 72h. (A) Incubation with ^3^H-ManNAc resulted in a significant reduction of incorporated ManNAc in the TGFBR2 clones #5 and #22 but not in the parental HCT116-Tet-On cell line. (B) Incorporation of ^3^H-L-fucose was slightly reduced in presence of dox in both TGFBR2 clones in contrast to HCT116-Tet-On cells. Values represent the mean of three independent experiments ±S.D.

Since we did not detect any changes in the steady state levels of cell surface proteins by Lectin-FACS analysis (Figure S2) and Lectin-Western blotting (Figure S3), we performed radioactive labeling experiments using two independent ^3^H-labeled monosaccharides, ManNAc and L-fucose. With this approach we focused our measurements on newly synthesized glycoproteins. In initial experiments, different time periods (24h, 48h and 72h) were examined. The incorporation of ^3^H-ManNAc, a precursor of sialic acid, increased over time with a peak at about 72 h. Upon dox exposure and addition of TGF-ß1 for 72 h, a significant reduction of incorporated ^3^H-ManNAc occurred in both TGFBR2 clones but not in the parental Tet-On cell line (Figure 4A). Therefore, we analyzed all 20 known sialyltransferases and two sialidases (Neu1 and Neu3) in real-time RT-PCR at 24h, 48h and 72h after induction (Table S2). However, we were not able to detect any changes in the mRNA expression levels (Figure S4). Re-expression of TGFBR2 led to a significant decrease in protein fucosylation in one of both clones using ^3^H-L-fucose (Figure 4B). These results suggest that TGFBR2 regulates sialylation of *de novo* proteins.

### ß1-integrin Expression and Sialylation

**Figure 5:**
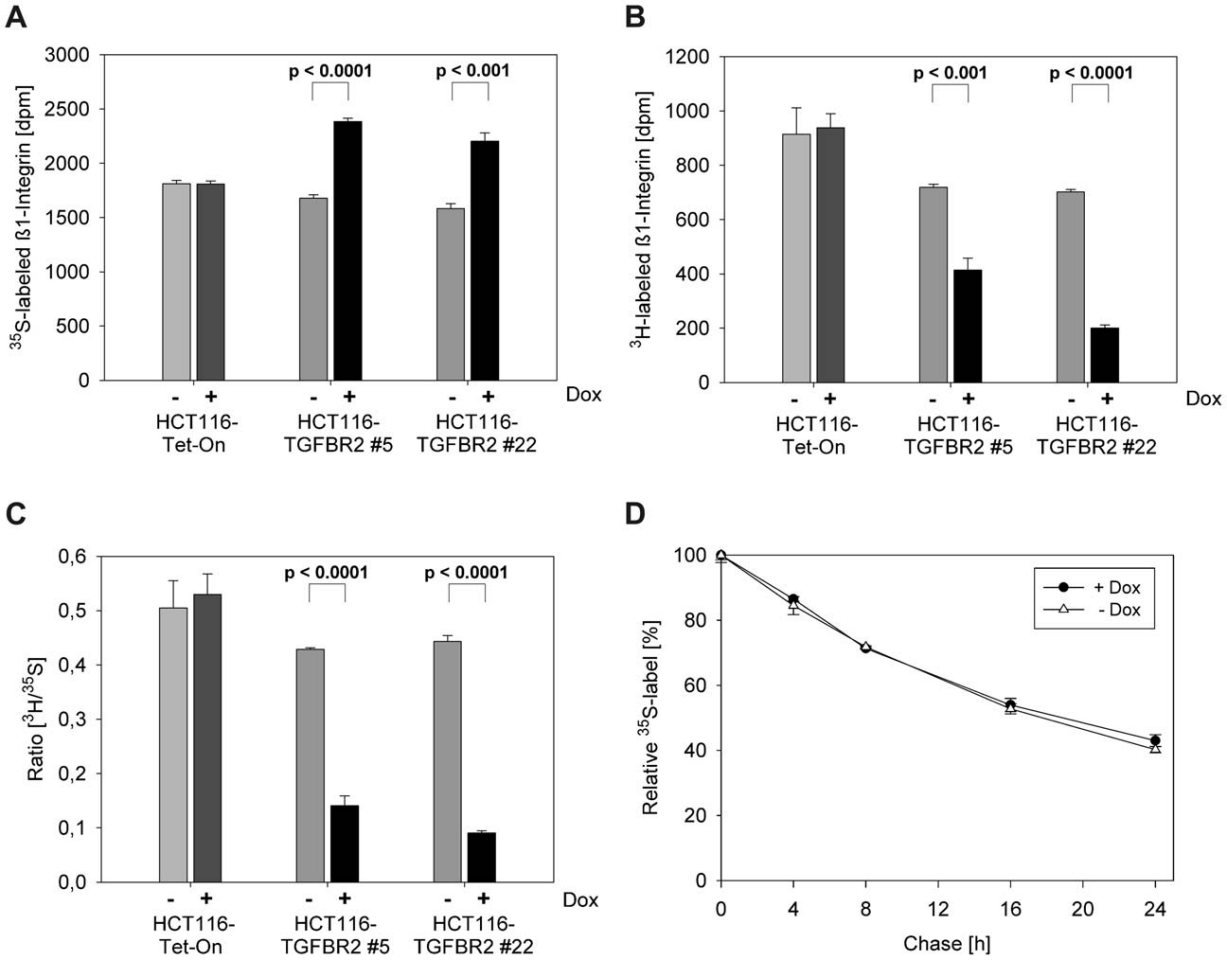
Analysis of ß1-integrin using radioactive labeling. (A–C) Dual labeling of cells with ^3^H-ManNAc and ^35^S-L-methionine was performed in presence and absence of dox (0.5 µg/ml) and in the presence of TGF-ß1 (10 ng/ml) for 72 h. Metabolic labeling resulted in elevated levels of ß1-integrin protein (A) and a significant reduction of incorporated ^3^H-ManNAc (B) in the TGFBR2 clones #5 and #22 but not in the parental HCT116-Tet-On cell line. (C) Normalizing of ^3^H-ManNAc incorporation into ß1-integrin to newly synthesized ^35^S-protein content (Ratio [^3^H/^35^S]). (D) Pulse-chase experiment was performed using ^35^S-L-methionine for 72 h (pulse) and immunoprecipitation at five different time points (chase). Values represent the means of three independent experiments ±S.D.

Since it is known that ß1-integrin is a highly sialylated protein whose expression is altered by TGF-ß1 [38], we next examined whether ß1-integrin sialylation might be affected by TGFBR2 expression and signaling. Based on our observation that TGFBR2 appears to modulate only sialylation of *de novo* proteins, we performed radioactive dual labeling experiments (^3^H-ManNAc and ^35^S-L-methionine) to determine the incorporation of sialic acid and as a control the synthesis of ß1-integrin in TGFBR2-induced cells (Figure 5). After labeling for 72h, the cells were harvested and ß1-integrin was immuno-precipitated. While reconstituted TGFBR2 signaling led to increased expression of ß1-integrin mRNA (2-fold) (not shown) and protein as determined by metabolic labeling (Figure 5A), the incorporation of ManNAc showed a TGFBR2-dependent decrease (Figure 5B). By normalizing the sialic acid incorporation to ß1-integrin synthesis the effect of TGFBR2 on ß1-integrin could be illustrated more strikingly, shown in Figure 5C. In order to determine whether the effect of TGFBR2 on ß1-integrin expression is due to an effect of altered sialylation on ß1-integrin stability, a pulse-chase experiment was performed. As indicated in Figure 5D the half-life of ß1-integrin protein was about 16 h and this turnover rate remained unchanged in the presence or absence of TGFBR2 expression. Overall, these data suggest that TGFBR2 signaling regulates the sialylation of *de novo* proteins in general and of ß1-integrin in particular without affecting its turnover.

## Discussion

In previous work we have demonstrated that transient transfection of *TGFBR2* into HCT116 cells, deficient for this receptor, led to alterations of cell surface glycosylation in these MSI tumor cells [30]. In order to overcome the transient nature of this expression system and to allow for a more detailed analysis of these TGFBR2-mediated glycan alterations and its associated downstream signaling effects, we have herein generated the HCT116-TGFBR2 MSI tumor cell line model system. It is important to note that results obtained from this TGFBR2-reconstituted system should reflect the inverse situation of the TGFBR2-deficient status in MSI primary colorectal tumors. Our experimental system carries a variety of salient features and has several advantages. For example, a single copy of the *TGFBR2* transgene is integrated into the MSI cancer cell line genome. Since expression of this transgene can be induced and regulated by dox, physiological levels of the encoded wildtype TGFBR2 protein can be achieved. In terms of time-scale analysis the consequences of short-term and long-term TGFBR2 expression and signaling on the glycobiology in MSI tumor cells can be easily determined. Furthermore, the single *TGFBR2* integration site in two independent HCT116-TGFBR2 cell clones has been identified at the nucleotide level and these clones allow reversible and persistent inducibility of target gene expression in an isogenic background. Moreover, this model cell line is particularly apt for large-scale production and isolation of altered glycoproteins and identification of derived glycopeptides that provide a source of novel targets suitable for MSI tumor diagnostics and therapy. Finally, the identified genomic loci can be re-targeted by any gene of interest when using the HCT116-HygTK master cell line and therefore provides a versatile tool for analyzing the functional consequences and biological relevance of a given MSI tumor-associated mutation.

In the present work we have used this HCT116-TGFBR2 model system to characterize TGFBR2-dependent alterations of protein glycosylation in MSI tumor cells. Since changes in the sialylation and fucosylation of glycoproteins are observed in many cancers, we particularly focused on these cancer-associated glycan modifications [39]. Initially, we did not detect any alterations of overall cell surface protein glycosylation by Lectin-FACS analysis as has been seen previously in our transient expression system [30]. This might be due to the TGFBR2 expression level conferred by a single copy transgene in HCT116-TGFBR2 cells which is far below the expression level of transiently transfected cells and hence minor glycosylation changes might have escaped detection by Lectin-FACS analysis. However, instead of detecting gross changes of steady state protein glycosylation levels, our metabolic labeling experiments indicate that the sialylation of *de novo* glycoproteins appears to be regulated by TGFBR2 signaling in this MSI tumor cell model system. These results not only confirm the TGFBR2-dependent decreased binding of the plant lectin SNA to sialylated cell surface glycoproteins initially observed in our previous work [30], but also argue in favor of sialylation of proteins being a dynamically regulated process modulated by a major signaling pathway in colon cancer cells.

Increased sialylation is known to occur frequently in tumor cells and correlates with their metastatic behavior [40]. The decreased sialylation upon TGFBR2 reconstitution in our MSI cell line model system supports this notion because it inversely reflects the loss of TGFBR2 function during MSI tumorigenesis. It has been reported that sialic acids may profoundly influence protein function. For example, membrane proteins like Fas death receptor, EGFR and ß1-integrin are known to exhibit variant sialylation that can affect chemo-sensitivity, metastasis and migration of colon tumor cells [27], [41], [42]. In our MSI tumor cell line model system, sialylation of ß1-integrin turned out to be modulated by TGFBR2 expression and signaling and was dependent on *de novo* protein synthesis. Colon tumor tissues exhibit hyper-sialylated ß1-integrin in contrast to normal tissues and it has been suggested that hyper-sialylation of ß1-integrin may contribute to cancer progression in colon adenocarcinoma [27]. This is consistent with our findings that reconstituted TGFBR2 expression in an MSI tumor cell line leads to less sialic acid incorporation on ß1-integrin, while the TGFBR2-deficient tumor exhibits increased sialylation. As an adhesion protein, ß1-integrin plays an important role in cancer metastasis. Sialylation of ß1-integrin in colon carcinoma cells has also been shown to block cell adhesion to galectin-3 and to protect against galectin-3-induced apoptosis [43].

Studies in different cell lines have shown that TGF-ß1 can regulate ß1-integrin expression at the transcriptional and translational level [44]. At the molecular level, TGF-ß1 has been shown to activate Ras and causing changes in glucosamine incorporation thereby providing a link between glycosylation and TGF-ß1 signaling [45]. In particular, oncogenic H-Ras has been reported to upregulate ß-galactoside α2,6-sialyltransferase 1 (ST6Gal1), one enzyme catalyzing the transfer of sialic acid, and subsequently to increase ß1-integrin sialylation causing up-regulated cell motility [27]. When we analyzed all 20 known human sialyltransferases and sialidases by real-time RT-PCR analysis at different time points, mRNA levels remained unaffected by reconstituted TGFBR2 signaling. One might speculate that regulation occurs at the protein level by altering enzymatic activity [46], [47]. A different putative mechanism may be the availability of sialyltransferase substrates. In colon cancer it has been shown that other glycosyltransferases, such as the beta1,4-*N*-acetylgalactosaminyltransferase, can compete with the sialyltransferases leading to altered glycan structures, including expression of the sialyl-Le^x^ and -Le^a^ antigens [48], [49]. The lack of transcriptional upregulation of ST6Gal1 expression despite TGFBR2-dependent variant ß1-integrin glycosylation in our experimental system raises some interesting questions. For example, are there Ras-independent mechanisms eventually altering cell surface protein sialylation pattern? Since about 40–50% of colorectal tumors have mutant Ras genes and about 90% of colorectal tumors have up-regulated ST6Gal1 activity and 70% of colorectal cancers are positive for the α2,6 sialic acid modification added by ST6Gal1 [50], Ras-independent but TGFBR2-mediated mechanisms of glycan alterations appear most likely. Moreover, is normal Ras signaling and hence downstream target gene transcription impaired in our model cell line? We know that these cells carry an oncogenic G13D *K-Ras* gene but are wildtype for *H-Ras*. Since ST6Gal1-associated ß1-integrin glycosylation has been reported to be regulated by H-Ras upon TGF-ß1 signaling [45], this molecular mechanism seems unlikely in our model system. Further, it has been described that the various *Ras* genes are very similar but eliciting different biological functions through their hyper-variable protein domain and localization within the cell [51], [52].

Here, we show for the first time the direct link between TGFBR2 expression and ß1-integrin protein upregulation and decreased sialylation. It has been known that TGF-ß1 leads to altered ß1-integrin expression [38], [44] and Ras-dependent glycan change of ß1-integrin [45]. However, it cannot be excluded that TGF-ß1 signaling might bypass TGFBR2. It has been described that TGF-ß1 may bind other cell surface proteins like Hyal-2 leading to a different signaling pathway as shown by Hsu *et al.* [53]. Further, it has been reported that in MSI CRC cell lines, that harbor inactivated TGFBR2, TGF-ß1 can induce growth inhibition and therefore bypass TGFBR2 [54], [55], suggesting that TGF-ß1 can induce other signaling pathways. This is supported by our observation that parental TGFBR2-deficient HCT116 cells as well as uninduced (-dox) HCT116-TGFBR2 cells showed increased basal pSMAD2 level when exposed to exogenous TGF-ß1 (Figure 3A). Overall, our approach directly addresses TGFBR2 signaling.

Protein sialylation plays a role in protein turnover and stability [56], [57] thereby affecting its cellular function. For this reason we investigated the effect of TGFBR2 on ß1-integrin stability by a pulse-chase experiment. However, the half-life of ß1-integrin was not altered in TGFBR2 expressing or deficient cells. The half-life has been approximately 16 h, as reported previously [58], [59]. Thus TGF-ß1-induced sialylation changes do not influence the stability of ß1-integrin.

Besides the prominent effects of TGF-ß1 signaling on protein sialylation, we also observed decreased protein fucosylation upon TGF-ß1 signaling, but to a lesser extent and only in one of both cell clones. TGFBR2 may therefore be a putative player in the regulation of protein fucosylation. Since fucosylation has been recently described as a biomarker in colorectal cancer [60], further investigations should follow to confirm our observation.

Our isogenic MSI tumor cell line model system provides the basis to analyze the signaling mechanisms that contribute to the glycobiology of MSI tumor cells and to identify the altered glycan structures. This may provide a novel source of MSI tumor-specific glycopeptides potentially useful for diagnostic and therapeutic applications.

## Supporting Information

Figure S1 **Proliferation assay and morphology of TGFBR2 clones**. (A) Growth of parental HCT116-Tet-On cells was compared with HCT116-TGFBR2 #5 cells in the presence or absence of dox and TGF-ß1. Results represent the mean of three independent experiments ±S.D. (B) Morphology of HCT116-Tet-On cells and both HCT116-TGFBR2 clones grown under different conditions for 48 h (magnification 40x).
(TIF)

Figure S2 **Lectin-FACS analysis.** Experiments were performed as described previously [30]. (A) Representative FACS analysis of the HCT116-Tet-On and HCT116-TGFBR2 #5 cells using biotinylated SNA. Cells were grown in the presence or absence of dox (0.5 µg/ml) for 72 h. (B) Panel of biotinylated plant lectins (Vector Laboratories) used for FACS analysis. Values represent the Y geometric mean fluorescence intensities. FSC, forward scatter; SSC, side scatter; FL1-H EGFP; FL2-H, streptavidin-PE (Sigma); nd, not determined; PSA, *Pisum sativum* agglutinin; JAC, Jacalin; DBA, *Dolichos biflorus* agglutinin; SNA, *Sambucus nigra* agglutinin; ConA, Concanavalin A; PHA-E, *Phaseolus vulgaris* erythroagglutinin; PHA-L, *Phaseolus vulgaris* leukoagglutinin; MAA-I, *Maackia amurensis* agglutinin-I; PNA, Peanut agglutinin; WGA, Wheat germ agglutinin.
(TIF)

Figure S3 **Lectin-Western blotting.** Cell lysates of HCT116-Tet-On and HCT116-TGFBR2 cells grown in the presence (+) and absence (-) of dox were separated by SDS-PAGE. Glycosylated proteins were detected by biotinylated SNA (A), MAA-I (B) and ConA (C) using streptavidin-HRP (SouthernBiotech).
(TIF)

Figure S4 **Expression analysis of sialyltransferases and sialidases.** Real-time RT-PCR analysis of sialyltransferase and sialidase transcripts that could be amplified in HCT116-TGFBR2 cells (24 h). Bars represent fold expression of mRNA in dox-treated versus untreated cells. Similar results were obtained after 48 h and 72 h. No significant changes (< 0.5- or > 2-fold induction) were observed.
(TIF)

Table S1 **Primer sequences.** For nrLAM-PCR modified primers were used: Biotin (B) and phosphate (P) modifications at the 5′ end and dideoxynucleotide (DDCLCI) modification at the 3′ end. Restriction sites in the cloning primers are indicated by italic letters.
(DOCX)

Table S2 **Sialyltransferase and sialidase specific primer sequences.** The upper sequence represents the forward primer whereas the lower sequence displays the reverse primer.
(DOCX)
