# Supplementary figures and images for: Transforming Growth Factor Beta Receptor 2 (TGFBR2) Changes Sialylation in the Microsatellite Unstable (MSI) Colorectal Cancer Cell Line HCT116
Source: PLoS One. 2013 Feb 27;8(2):e57074. doi: 10.1371/journal.pone.0057074 (PMC3584148; doi:10.1371/journal.pone.0057074)

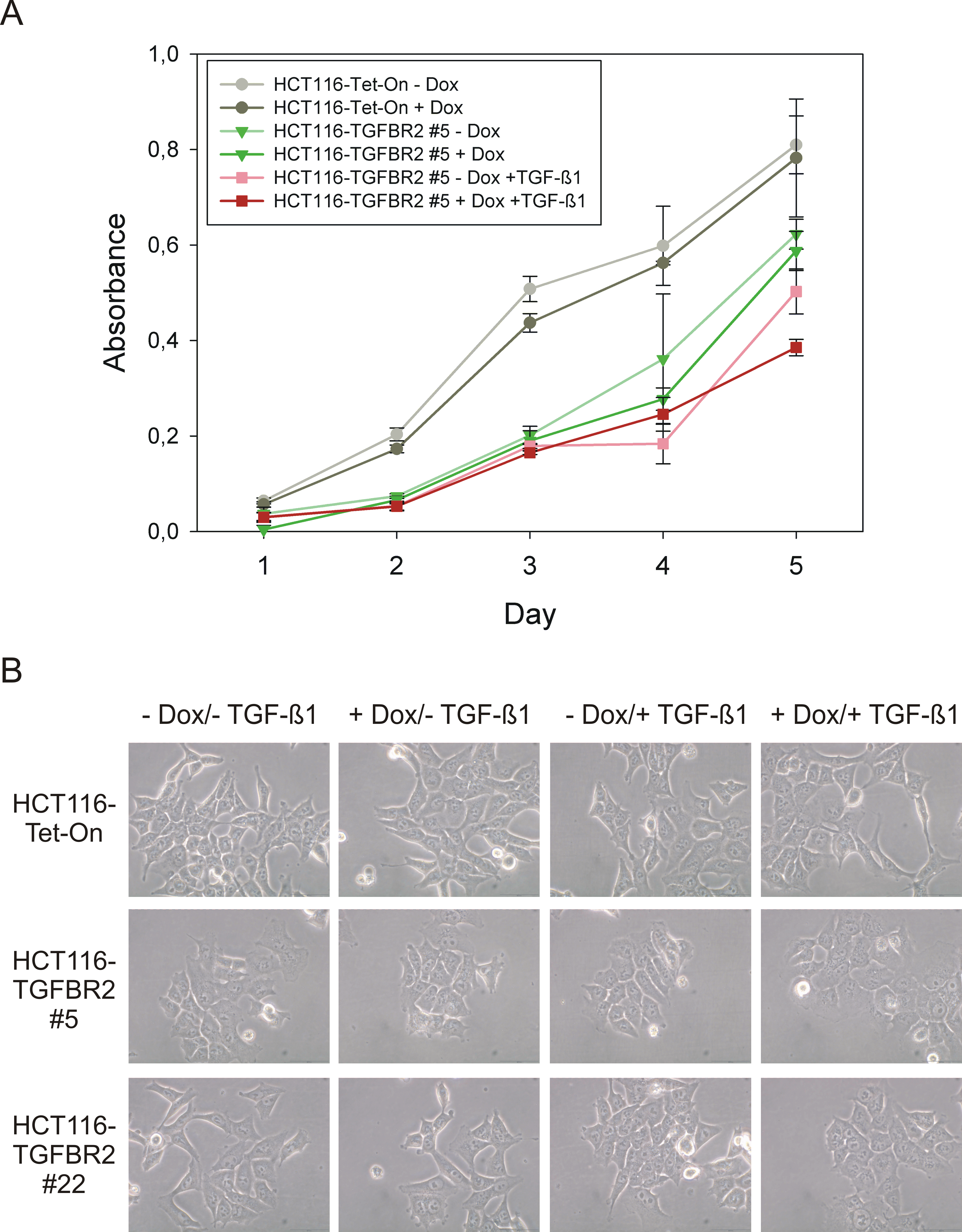

Supplement: Figure S1 — Proliferation assay and morphology of TGFBR2 clones. (A) Growth of parental HCT116-Tet-On cells was compared with HCT116-TGFBR2 #5 cells in the presence or absence of dox and TGF-ß1. Results represent the mean of three independent experiments ±S.D. (B) Morphology of HCT116-Tet-On cells and both HCT116-TGFBR2 clones grown under different conditions for 48 h (magnification 40x). (TIF) [file pone.0057074.s001.tif]

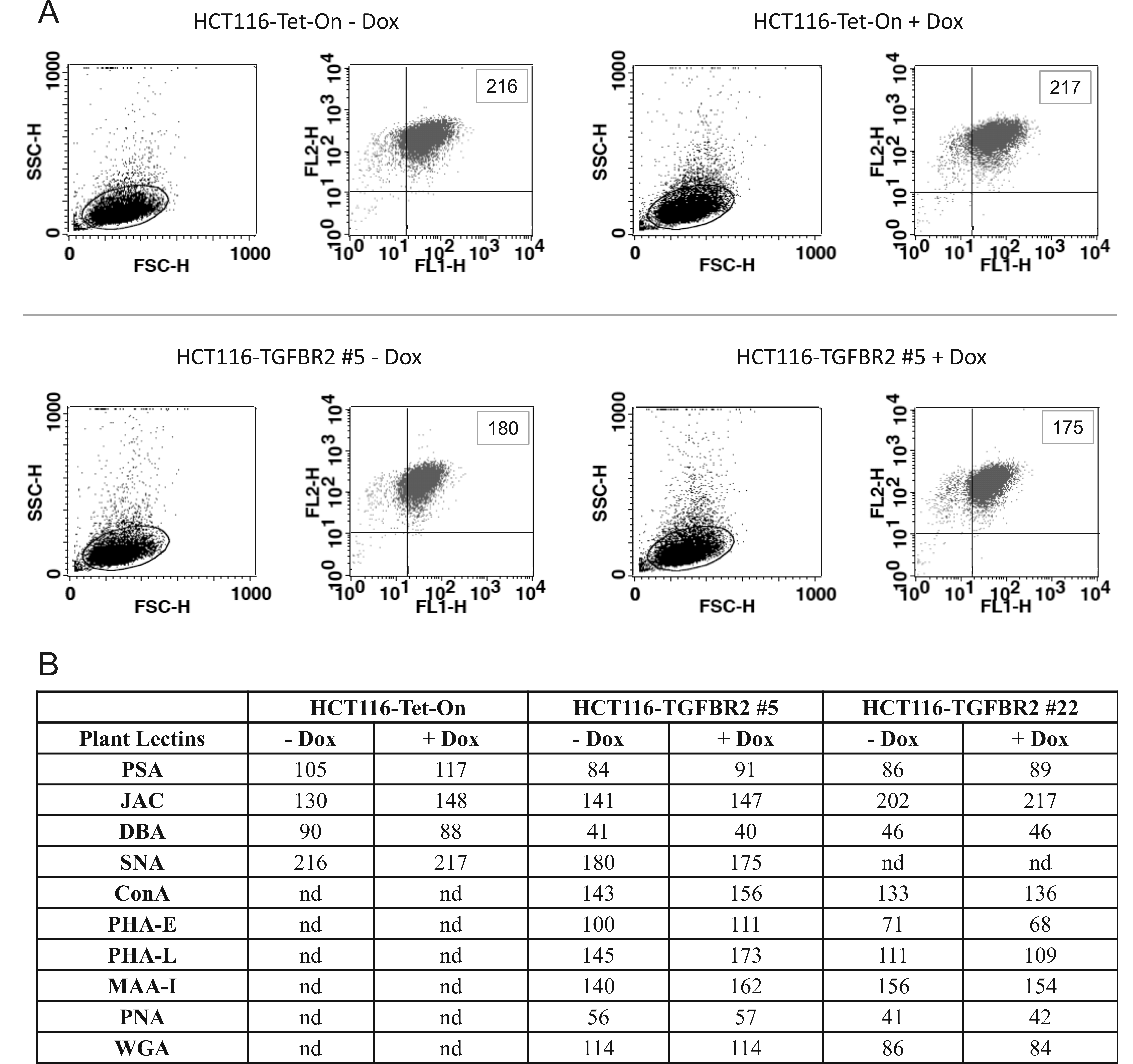

Supplement: Figure S2 — Lectin-FACS analysis. Experiments were performed as described previously [30]. (A) Representative FACS analysis of the HCT116-Tet-On and HCT116-TGFBR2 #5 cells using biotinylated SNA. Cells were grown in the presence or absence of dox (0.5 µg/ml) for 72 h. (B) Panel of biotinylated plant lectins (Vector Laboratories) used for FACS analysis. Values represent the Y geometric mean fluorescence intensities. FSC, forward scatter; SSC, side scatter; FL1-H EGFP; FL2-H, streptavidin-PE (Sigma); nd, not determined; PSA, Pisum sativum agglutinin; JAC, Jacalin; DBA, Dolichos biflorus agglutinin; SNA, Sambucus nigra agglutinin; ConA, Concanavalin A; PHA-E, Phaseolus vulgaris erythroagglutinin; PHA-L, Phaseolus vulgaris leukoagglutinin; MAA-I, Maackia amurensis agglutinin-I; PNA, Peanut agglutinin; WGA, Wheat germ agglutinin. (TIF) [file pone.0057074.s002.tif]

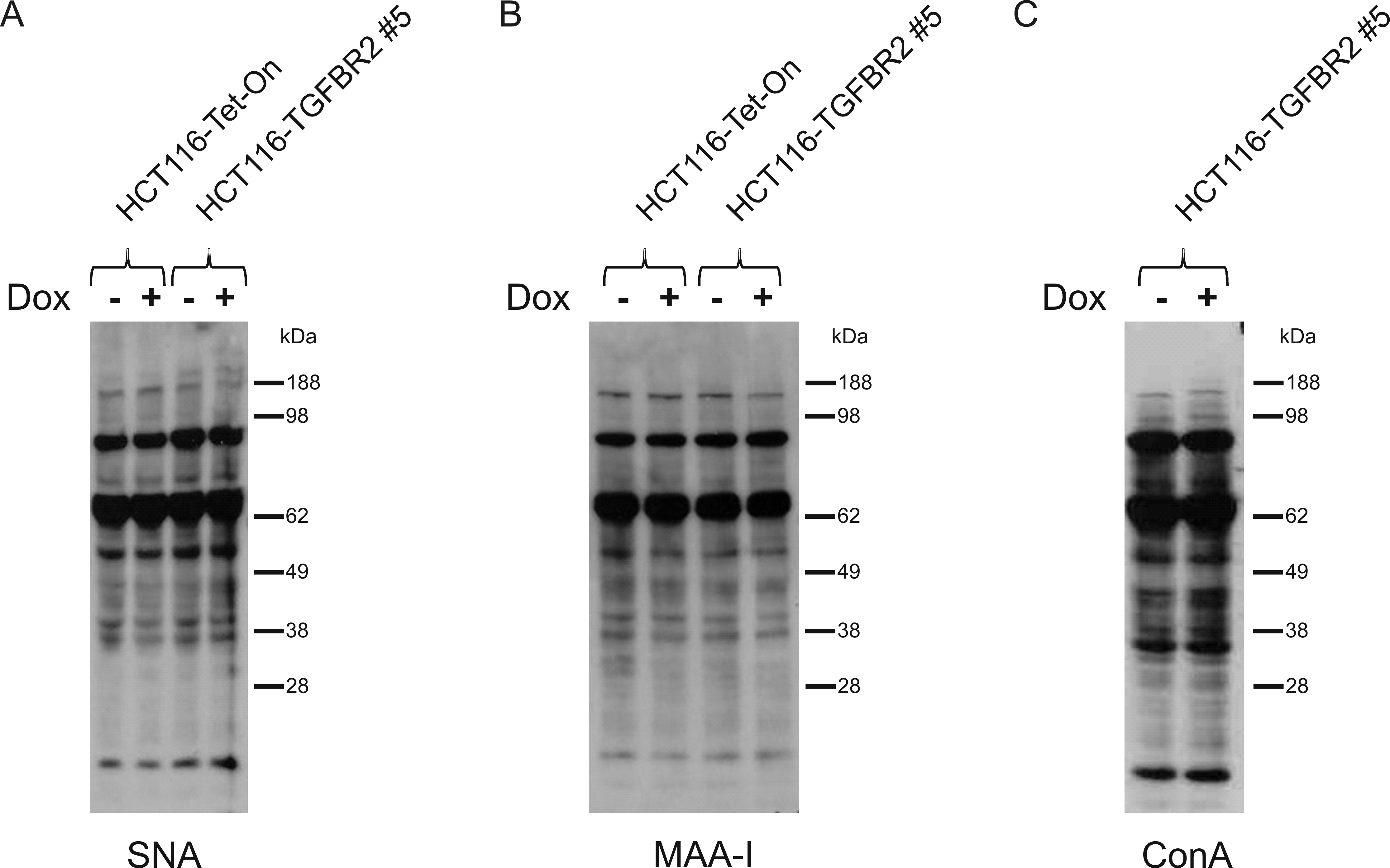

Supplement: Figure S3 — Lectin-Western blotting. Cell lysates of HCT116-Tet-On and HCT116-TGFBR2 cells grown in the presence (+) and absence (-) of dox were separated by SDS-PAGE. Glycosylated proteins were detected by biotinylated SNA (A), MAA-I (B) and ConA (C) using streptavidin-HRP (SouthernBiotech). (TIF) [file pone.0057074.s003.tif]

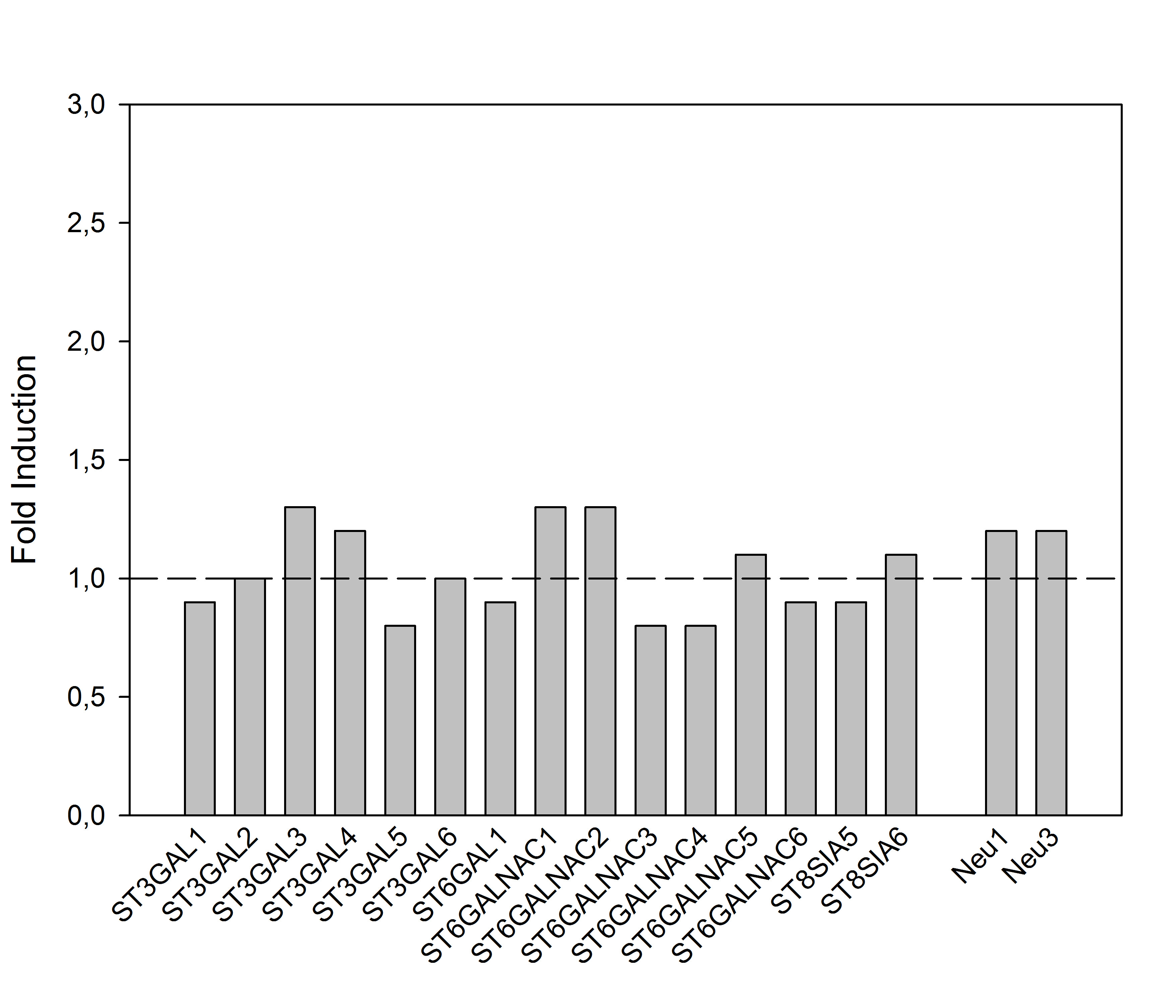

Supplement: Figure S4 — Expression analysis of sialyltransferases and sialidases. Real-time RT-PCR analysis of sialyltransferase and sialidase transcripts that could be amplified in HCT116-TGFBR2 cells (24 h). Bars represent fold expression of mRNA in dox-treated versus untreated cells. Similar results were obtained after 48 h and 72 h. No significant changes (< 0.5- or > 2-fold induction) were observed. (TIF) [file pone.0057074.s004.tif]
