# Supplementary material for: Transforming Growth Factor Beta Receptor 2 (TGFBR2) Changes Sialylation in the Microsatellite Unstable (MSI) Colorectal Cancer Cell Line HCT116
Source: PLoS One. 2013 Feb 27;8(2):e57074. doi: 10.1371/journal.pone.0057074 (PMC3584148; doi:10.1371/journal.pone.0057074)
Supplement: Table S1 — Primer sequences. For nrLAM-PCR modified primers were used: Biotin (B) and phosphate (P) modifications at the 5′ end and dideoxynucleotide (DDCLCI) modification at the 3′ end. Restriction sites in the cloning primers are indicated by italic letters. (DOCX) [file pone.0057074.s005.docx]

**Table S1. Primer sequences.**

| Cloning | Sequence [5´-3´] |
| --- | --- |
| TGFBR2_*EcoRI*_F1 | AG*GAATTC*GCCATGGGTCGGGGGCTG |
| TGFBR2_*NotI*_R1 | GGGG*GCGGCCGC*CTATTTGGTAGTGTTTAGGGAG |
| Sequencing |  |
| TGFBR2_for_577 | AGGAAGTCTGTGTGGCTGTATG |
| TGFBR2_3UTR_rev1 | GAGTGACCCCCTTGGTTTTC |
| S2F_seq_R1 | AACAAATTGGACTAATCCGGA |
| nrLAM-PCR |  |
| P1 | B-TCTTGCAGTTGCATCCGACT |
| P2 | P-CCTAACTGCTGTGCCACTGAATTCAGATCTCCCGGGTC-DDCLCI |
| P3 | B-GTGGTCTCGCTGTTCCTT |
| P4 | GATCTGAATTCAGTGGCACAG |
| P5 | GTCTCCTCTGAGTGATTGAC |
| Integration Site |  |
| C1orf159_F1 | CTGGAGGGTCGTGTCCTTAG |
| U5_R1 | GGAACAGCGAGACCACAAGT |
| mCherry_F2 | GCCTACAACGTCAACATCAAG |
| C1orf159_R2 | GTCCAGTCCCTCCCTTTCTC |
| ALDH1L1_In4_F1 | AGGAGCTGAGGAGGACGAG |
| ALDH1L1_In4_R1 | CACAGGCAGAGGTGCTGTC |
| Real-time RT-PCR |  |
| GAPDH_F1 | AGCCACATCGCTCAGACAC |
| GAPDH_R1 | GCCCAATACGACCAAATCC |
| HMBS_F1 | CACCACAGGGGACAAGATTC |
| HMBS_R1 | GTGAACAACCAGGTCCACTTC |
| TGFBR2_F1 | CGGCTCCCTAAACACTACCAA |
| S2F_seq_R1 | AACAAATTGGACTAATCCGGA |
| TGFBR2_F1 | CGGCTCCCTAAACACTACCAA |
| TGFBR2_3UTR_R2 | CTTCCTGCTGCCTCTGTTCTT |
| TGFBR13_F1 | TGTCTACTCCATGGCTCTGGT |
| TGFBR2_R_1769 | CCTTGGAACCAAATGGAGG |
| SMAD7_Ex1_for1 | CTCGGAAGTCAAGAGGCTGT |
| SMAD7_Ex1_rev1 | GCAGAGTCGGCTAAGGTGAT |
| SERPINE_Ex4_for1 | CAACTTGCTTGGGAAAGGAGC |
| SERPINE_Ex4_rev1 | AGTCGGGGAAGGGAGTCTTC |
