# Supplementary material for: Transforming Growth Factor Beta Receptor 2 (TGFBR2) Changes Sialylation in the Microsatellite Unstable (MSI) Colorectal Cancer Cell Line HCT116
Source: PLoS One. 2013 Feb 27;8(2):e57074. doi: 10.1371/journal.pone.0057074 (PMC3584148; doi:10.1371/journal.pone.0057074)
Supplement: Table S2 — Sialyltransferase and sialidase specific primer sequences. The upper sequence represents the forward primer whereas the lower sequence displays the reverse primer. (DOCX) [file pone.0057074.s006.docx]

**Table S2. Sialyltransferase and sialidase specific primer sequences.**

| **Name of Sialyltransferase** | ***Gene*** | **Sequence [5´-3´]** |
| --- | --- | --- |
| ST3 beta-galactoside alpha2,3-sialyltransferase 1 | *ST3GAL1* | GACTTGGAGTGGGTGGTGAG GGAACCGGGATGTAGGTGT |
| ST3 beta-galactoside alpha2,3-sialyltransferase 2 | *ST3GAL2* | TGCATGTGTGTGATGAGGTG  TGATGTGGGCCTCGAAGT |
| ST3 beta-galactoside alpha2,3-  sialyltransferase 3 | *ST3GAL3* | GCACCCCTGCACTACTATGAG  GCTGGATATTGTGCGTCCA |
| ST3 beta-galactoside alpha2,3-  sialyltransferase 4 | *ST3GAL4* | TGAGCAGATCACGCTCAAGT  AGGACGTGAGGTTCTTGATAGC |
| ST3 beta-galactoside alpha2,3-  sialyltransferase 5 | *ST3GAL5* | CTGCCTTTGACATCCTTCAGT  CGATTGTGGGGACGTTCTTA |
| ST3 beta-galactoside alpha2,3-  sialyltransferase 6 | *ST3GAL6* | AAAAAGTGTGTGGTGGTTGGT  CCTAAAACAGGACCATTATTCATTC |
| ST6 beta-galactosamide alpha2,6-sialyltranferase 1 | *ST6GAL1* | AGGTGTGCTGTTGTGTCGTC  TAAACCTCAGGACTGCGTCA |
| ST6 beta-galactosamide alpha2,6-sialyltranferase 2 | *ST6GAL2* | TCACTGTATAAAGACGTCATTTTGGT  GAACAGGTTGTAATCCGGTTTTT |
| ST6 (alpha-*N*-acetyl-neuraminyl-2,3-beta-galactosyl-1,3)-*N*-acetylgalactosaminide  alpha2,6-sialyltransferase 1 | *ST6GALNAC1* | CGAAATAGGAGGCCTTCAGA  AGAGAGTGAGGTTGGGCAGA |
| ST6 (alpha-*N*-acetyl-neuraminyl-2,3-beta-galactosyl-1,3)-*N*-acetylgalactosaminide  alpha2,6-sialyltransferase 2 | *ST6GALNAC2* | TGTCCCTGAGGGCCTAGATA  CCGGATGTAGCAGCTTGAAT |
| ST6 (alpha-*N*-acetyl-neuraminyl-2,3-beta-galactosyl-1,3)-*N*-acetylgalactosaminide  alpha2,6-sialyltransferase 3 | *ST6GALNAC3* | TTACTGTGATGGAGTTTTTAAGAAGG  CCACCCTGTGCTGAGATATG |
| ST6 (alpha-*N*-acetyl-neuraminyl-2,3-beta-galactosyl-1,3)-*N*-acetylgalactosaminide  alpha2,6-sialyltransferase 4 | *ST6GALNAC4* | AGATCTTCCAGGACGAGACG  GACCACGATCTCCTCACACA |
| ST6 (alpha-*N*-acetyl-neuraminyl-2,3-beta-galactosyl-1,3)-*N*-acetylgalactosaminide  alpha2,6-sialyltransferase 5 | *ST6GALNAC5* | AGGAGACTGGCAAAGACAGG  GTCACAGAGCTCCAGTGCAA |
| ST6 (alpha-*N*-acetyl-neuraminyl-2,3-beta-galactosyl-1,3)-*N*-acetylgalactosaminide  alpha2,6-sialyltransferase 6 | *ST6GALNAC6* | GCAATTTGACGACCTCTTCC  TGTGCTCAACCACGAATGA |
| ST8 alpha-*N*-acetyl-neuraminide alpha2,8-sialyltransferase 1 | *ST8SIA1* | CAACTTACTCTCTCTTCCCACAGG  TGACAAAATTTGCTTCATCTATTTG |
| ST8 alpha-*N*-acetyl-neuraminide alpha2,8-sialyltransferase 2 | *ST8SIA2* | CCTGAAGCACCACGTCAAC  TGTGGCCAGGGTATACATCA |
| ST8 alpha-*N*-acetyl-neuraminide alpha2,8-sialyltransferase 3 | *ST8SIA3* | CTTGGCCGGGAAATATAATG  TATTGCTGATGCAAGGGTGT |
| ST8 alpha-*N*-acetyl-neuraminide alpha2,8-sialyltransferase 4 | *ST8SIA4* | CGAAAGGCTGGCTCTTCA  CGTTCTGCATCTAAGAAACGAAG |
| ST8 alpha-*N*-acetyl-neuraminide alpha2,8-sialyltransferase 5 | *ST8SIA5* | CCAGCATCATCACAGAGAGG  GTACTTGACGCGGATGGAC |
| ST8 alpha-*N*-acetyl-neuraminide alpha2,8-sialyltransferase 6 | *ST8SIA6* | AATCCAAGCATCATAACTCTGAAA  AGGAAAATGCTGGCAGAAGA |

| **Name of Sialidase** | ***Gene*** | **Sequence [5´-3´]** |
| --- | --- | --- |
| Sialidase 1 (lysosomal sialidase) | *NEU1* | CGGGCTCTGGTATTCAGAAA  TGACCCCACTTCCGTAGC |
| Sialidase 2 (cytosolic sialidase) | *NEU2* | CCACCCACCAGGTTCAGT  TACAAGGGGCATGGGTTC |
| Sialidase 3 (membrane sialidase) | *NEU3* | GGATTGGGCAGTTGGTACAG  GAACAGGAACACACAACCACTC |
| Sialidase 4 | *NEU4* | CTCAGCCCTGACGACTCC  GTTCATGGACCGGTGCTC |
